# Evaluation of the antidepressant potential of Duloxetine, *Coffea canephora*, and *Nigella sativa* in a rat depression model

**DOI:** 10.1007/s11011-025-01718-3

**Published:** 2025-11-18

**Authors:** Enas S. Abdel-Baky, Shadia A. Radwan, Faten Mohamed Abdelhamid, Omnia N. Abdelrhman

**Affiliations:** Department of Biological and Geological Sciences, Faculty of Education, Ain Shams University, Cairo, Egypt

**Keywords:** Depression, Reserpine, Duloxetine, Green coffee, Black seeds

## Abstract

Depression is a complex disorder involving neurotransmitter imbalance, oxidative stress, and inflammation of the brain tissue. Traditional antidepressants like duloxetine (Cymbalta^®^) can produce side effects. Natural extracts like *Coffea canephora* (green coffee) and *Nigella sativa* (black seeds) have emerged as possible alternative therapies. The present study aims to evaluate the antidepressant efficacy of Cymbalta, green coffee, and black seeds in a reserpine-induced rat depression model. Thirty adult male albino rats were divided into five groups: (1) Control group, (2) Depression-induced group (reserpine 0.2 mg/kg for 14 days, followed by 0.1 mg/kg intraperitoneally), (3) Cymbalta-treated group (30 mg/kg orally for four weeks), (4) Green coffee-treated group (400 mg/kg orally for four weeks), and (5) Black seed-treated group (350 mg/kg orally for four weeks). Behavioral tests (FST, Y-maze), neurotransmitter (DA, 5-HT, NE), oxidative stress markers (MDA, NO, GSH), and neuroinflammatory cytokine levels (Iba-1) were analyzed. Cymbalta significantly decreased immobility time in FST and Y-maze, restored neurotransmitter balance, reduced oxidative stress, and mitigated neuroinflammation that induced by reserpine. Also, the green coffee and black seeds demonstrated antidepressant effects against the alterations induced by reserpine, the treatment with black seeds exhibiting superior neurochemical and antioxidant benefits compared with green coffee treatment. Cymbalta showed the greatest antidepressant action, but the green coffee and the black seeds presented good neuroprotective and antidepressant activities. These findings suggest that the natural compounds may serve as adjunctive therapies for depression.

**Graphical abstract:**

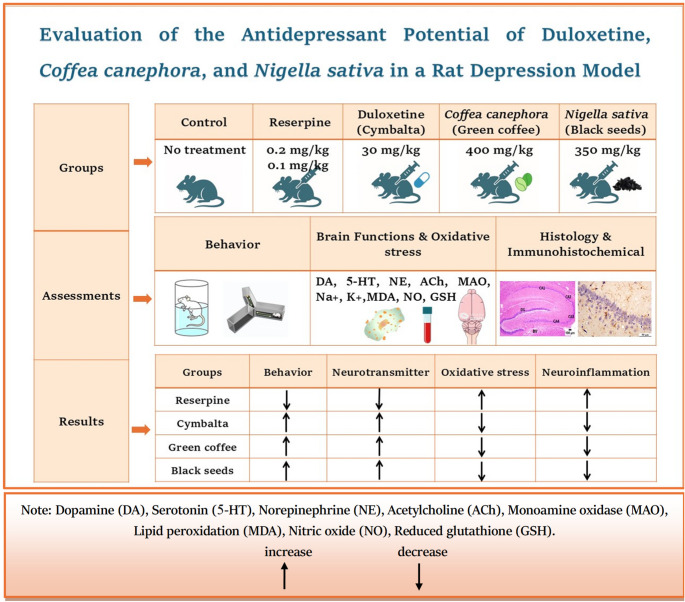

## Introduction

Depression is a serious mental illness affecting behavior, psychology, and physical well-being and has serious societal and economic consequences (Rana et al., 2020). According to estimates by the World Health Organization (WHO), depression is expected to become the globe’s greatest cause of disability by the year 2030 (Abdel-Rasoul et al., 2023). Depression has a close association with suicidal thoughts, cardiovascular diseases, compromised quality of life, higher morbidity and mortality rates, and compromised functioning and well-being at a societal level (Bhatt et al., 2023). It is considered as a complex disorder characterized by dysregulation of sympathetic nervous, endocrine, and immune systems (Ruiz et al., 2022). Monoamine neurotransmitters like dopamine (DA), serotonin (5-HT), norepinephrine (NE) and their metabolites have emerged from neurobiological and clinical studies as important in the pathophysiology of depression (Nakamura et al., 2022). Theoretical models dominating the present era hold that depressive illnesses stem from the dysregulation of single or multiple neurotransmitter systems in brain areas involved in mood modulation, including the cerebral cortex and hippocampus (Abdel-Rasoul et al., 2023).

Previous studies have identified various natural and synthetic compounds capable of inducing depressive-like behaviors in animal models. Reserpine, a drug used in the treatment of neuropsychiatric conditions and hypertension, has been used extensively to produce depressive symptoms in experimental studies (Abdel-Rasoul et al., 2023; Samad et al., 2021). The results of depression research illuminated the early application of reserpine as a drug used to create depression in experimental animal studies (Davies And Shepherd, 1955). Long-term reserpine administration is linked to brain monoamines depletion, thus triggering the development of depressive phenotypes (Strawbridge et al., 2023).

Duloxetine (Cymbalta^®^), a serotonin and norepinephrine reuptake inhibitor (SNRI), is used in preclinical antidepressant studies for treatment-resistant depression. It alleviates symptoms of depression, anxiety disorders, neuropathic pain, fibromyalgia, and chronic musculoskeletal pain, increases mood and reduces pain signals (Kato et al., 2022). Notwithstanding the development of numerous therapeutic regimens, including Cymbalta, a very high percentage of the patient populace registers suboptimum responses to traditional antidepressants. These challenges are usually explained by the gradual development of effects as well as the side effects and exorbitant expenditure involved in drug regimens, prompting noncompliance or drug discontinuation by the patient populace. As a result, interest has grown toward investigating alternative drugs featuring medicinal plants as a means of coping with the complex challenges of disease states caused by mental afflictions (Remali And Aizat, 2024).

Medical herbs are a rich reservoir of bioactive compounds featuring a plethora of therapeutic characteristics and properties for which many provide the inspiration for the development of medicinal drugs. These secondary metabolite-enriched phytochemicals have extensively been investigated for their medicinal uses as well as their capacity in the treatment of depression and other diseases (Nasim et al., 2022). Specifically, some plant-based interventions have proved to be effective in reducing depressive symptoms and have presented a less expensive and well-tolerated substitute for conventional antidepressants. For instance, green coffee and black seeds have surfaced as promising leads for natural antidepressants and present alternative options to current treatment regimens (Remali And Aizat, 2024).

Green coffee *Coffea canephora* (robusta), a commonly consumed beverage, has several benefits related to human health, including a lower risk of malignancies and depression (Hutachok et al., 2021). Its bioactive compounds primarily explain the above benefits as a result of their remarkable anti-inflammatory and antioxidant activities (Fernandes et al., 2021). The significant compounds involved in the above effects are chlorogenic acid, caffeic acid, pyrogallic acid, ferulic acid, and trigonelline, which explain their beneficial effects on both psychological and physical well-being (Socała et al., 2021). Recently, Mosalam et al. (2025) demonstrated that green coffee chlorogenic acids reversed cognitive deficits in behavioral tests, reduced neuroinflammation, and minimized neuronal damage in brain regions, confirming its antidepressant effects.

*Nigella sativa* or black seeds occupy a very important position in traditional medicine, especially in the context of Islamic civilization, as it has been a panacea for most of the diseases (Ahmad et al., 2013). The medicinal herb possesses a plethora of pharmacological effects ranging from antioxidant, anti-inflammatory, and immunomodulatory effects through neuroprotective and antitumor ones. It has also shown therapeutic effects on enhancing spermatogenesis in males, improving cognitive function, and conferring beneficial effects on rat and human models of lung and liver fibrosis and diabetic nephropathy (Imam et al., 2016). Kuzay et al. (2022) reported that black seeds thymoquinone ameliorated the changes occurred by reserpine in behavioral tests, reduced oxidative stress and increased neurotransmitters levels.

## Materials and methods

### Experimental animals

Thirty adult albino male rats (Rattus norvegicus) weighing about 140–160 g and aged between 4 and 5 months were obtained from Theodor Bilharz Research Institute, El-Giza, Egypt. The animals were maintained in clean plastic crates (three animals per crate) and given a rat pelleted conventional food supplemented with water ad libitum throughout the experimental phase. The animals were maintained under optimum hygienic conditions; under a light-dark cycle of 12 h, a temperature of 25 °C and a general humidity of 55 ± 5%. All rats were given two weeks of acclimation before the beginning of the experiment. All procedures were conducted according to ethical standards as approved by the Institutional Animal Ethics Committee of Ain Shams University (approval code: sci1332508001).

## Pharmacological and herbal materials

### Reserpine

was purchased from Thermo Fisher Scientific, USA (Kit: L03506.03; purity: 99%).

### Cymbalta

(Antidepressant drug) was obtained from Eli Lilly and Company Indianapolis, IN 46,285, USA.

### Green coffee and black seeds

were purchased from the local market Abu Auf in Cairo, Egypt.

### Plant extraction

According to Zygler et al. (2012), black seeds and green coffee beans were cleaned, dried, and ground into powder. Then, 100 g of the powder was subjected to Soxhlet extraction with 1000 mL of 70% ethanol (1:10 w/v) for 6 h. The final extract was filtered, concentrated via rotary evaporation at 40–45 °C, and heated at 40 °C to remove residual solvent then stored in light-resistant containers at 4 °C until use. Before oral administration, the extract was reconstituted in 0.9% saline and adjusted to the required dose (green coffee 400 mg/kg, black seeds 350 mg/kg).

### Induction of depression

A rat model of depression was established using reserpine according to Antkiewicz-Michaluk et al. (2014) and (Khadrawy et al., 2018).

#### Induction phase

 Reserpine was freshly prepared and dissolved in glacial acetic acid (1 µg/µl), then completed to 25 ml with distilled water. Rats received daily intraperitoneal injections (i.p.) of reserpine (0.2 mg/kg) for 14 consecutive days to induce a depressive-like state.

#### Maintenance phase

 Starting on day 15, the reserpine dose was reduced to 0.1 mg/kg and administered daily via i.p. injection for four weeks to maintain the depressive state.

### Experimental design

In line with the ARRIVE 2.0 guidelines, all thirty rats were randomly divided into five groups (*n* = 6) using a computer-generated sequence before any treatments began. Blinding was maintained by having independent researchers handle group labeling and treatment, while outcome assessors remained unaware of group assignments to minimize bias and ensure reproducibility. The timeline of the study showed in figure (1).

- **Group 1** (control group) served as a control and was fed standard food and allowed to drink fresh tap water throughout the study without any treatment.
- **Group 2** (depressed group): Rats were intraperitoneally (i.p.) administered reserpine (0.2 mg/kg b.wt.) for 14 days and then (0.1 mg/kg b.wt., i.p.) for four weeks (Antkiewicz-Michaluk et al., 2014).
- **In Group 3** (Cymbalta depressed group), the animals were injected i.p. with reserpine for 14 days and then treated with Cymbalta drug (30 mg/kg b.wt., orally) for four weeks (Ali et al., 2025).
- **In Group 4** (green coffee depressed group), the rats were treated with reserpine i.p. for 14 days and then with green coffee extract (400 mg/kg b.wt., orally) for four weeks (Molska et al., 2021; Yusni And Yusuf, 2022).
- ** In Group 5 **(black seeds depressed group), the rats were treated with reserpine i.p. for 14 days and then with black seeds (350 mg/kg b.wt., orally) for four weeks (Abdel-Rahman et al., 2013).

-
  **Fig. 1:**
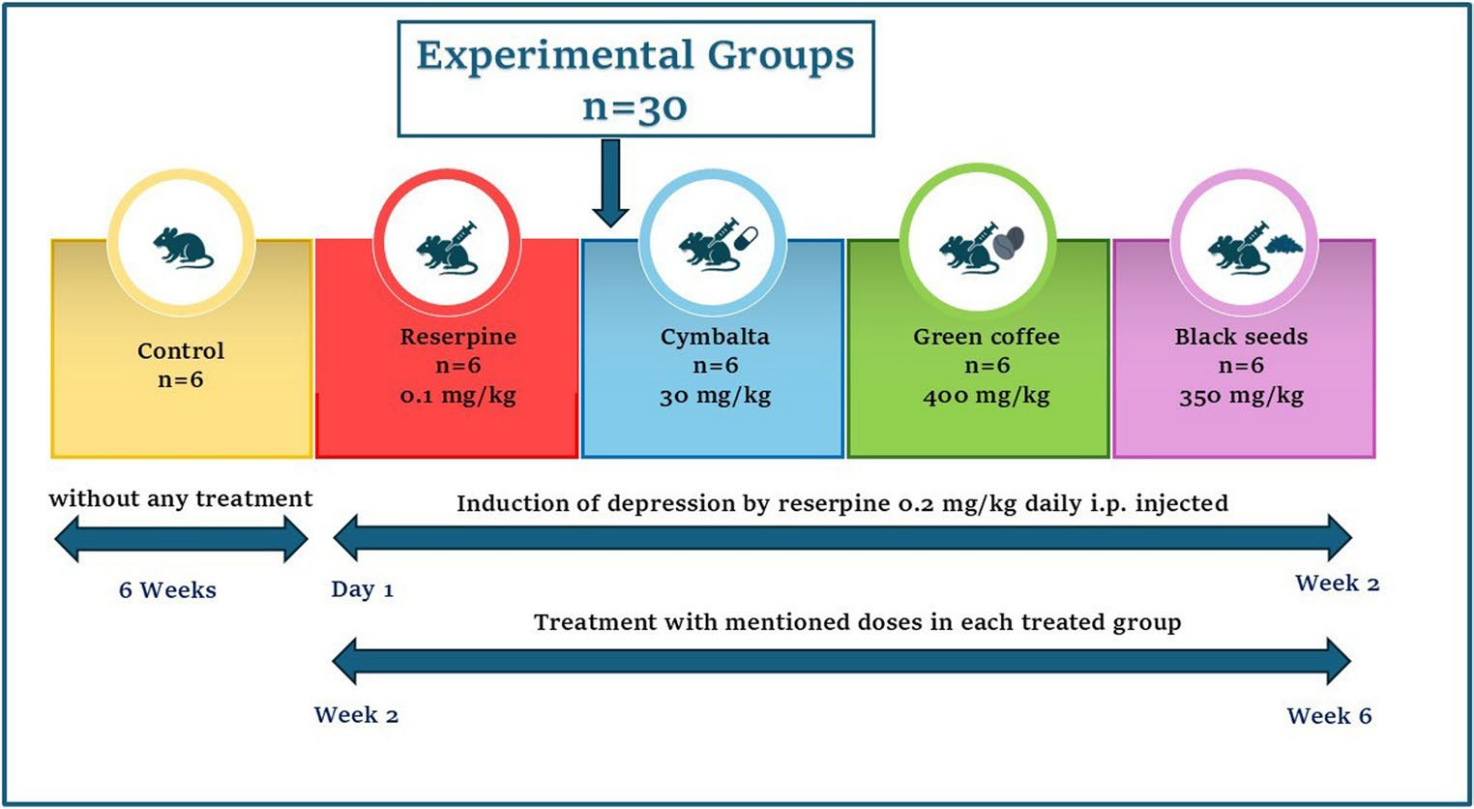
Timeline of the Study

### Behavioral assessments

Behavioral assessments were conducted with a 1-hour interval between each test to minimize potential interference from previous tests. 

### Forced swim test (FST)

The forced swim test (FST) was used to measure depression-like behavior in the rats. The first 2 min were an acclimatization period in the 6-minute test, and the immobility time was measured during the last 4 min. The immobility time was calculated by reducing the total mobility time from the 240-second test duration (Porsolt et al., 1977).

### Y-maze

The Y-maze test was used to measure spontaneous alternation behavior, an indicator of spatial working memory. Spontaneous alternation was characterized as successive entries into all three maze arms without repetition. The percentage of spontaneous alternation was measured via the following equation: [total number of arm entries – 2] × 100% (Prieur And Jadavji, 2019).

### Biochemical Preparation

Blood was collected from the retro-orbital plexus using a fine capillary tube then immediately transferred to serum separation tubes and left to clot at room temperature for 30 min, then centrifuged at 4000 rpm for 15 min at 4 °C. The resulting supernatant serum was stored at −20 °C or lower for biochemical analysis (Alayunt et al., 2023).

### Brain tissue Preparation

Rats were sacrificed, then the brains were carefully removed, rinsed with ice-cold saline to remove blood and debris then divided into two groups; the first brain tissues group were homogenized in Tris-HCl buffer (pH 7.4) at a 1:10 (w/v) ratio, followed by centrifugation at 5000 rpm for 10 min at 4 °C. The resulting supernatant was collected and stored at −70 °C for subsequent biochemical analyses (Khadrawy et al., 2018). The second brain tissues group were fixed in suitable fixative for histological & immunohistochemical analyses.

### Neurochemical measurements

#### Lipid peroxidation (MDA)

Malondialdehyde (MDA) content labelled as a marker of lipid peroxidation was quantified through the thiobarbituric acid reactive substances (TBARS) method. 200µL of homogenate react with 1mL TBARS in an acidic medium at temperature of 95 °C for 20 min to form thiobarbituric acid reactive substances. The absorbance of the pink complex at 532 nm was measured by a UV-visible spectrophotometer as previously described by Ruiz-Larrea et al. (1994).

### Nitric oxide (NO)

The level of nitrite was indirectly determined by employing 100µL of homogenate with 100 µL Griess reagent to measure nitrite levels. The purple azo dye obtained from this reaction with nitrite was subjected to measurement of absorbance at 540 nm (Montgomery And Dymock, 1961).

### Reduced glutathione (GSH)

Reduced glutathione (GSH) levels were determined quantitively by Ellman’s reagent method through spectrophotometry. When 100µL of homogenate and 100µL 5,5’-dithiobis (2-nitrobenzoic acid) (DTNB) react together, 2-nitro-5-thiobenzoic acid (TNB) having a yellow color is formed. The absorbance of TNB at 412 nm was determined (Beutler et al., 1963).

### Acetylcholine (ACh) activity

Measurements of acetylcholinesterase (ACh) activity were made through a spectrophotometric method. The method employs acetylthiocholine iodide as a substrate for ACh and is cleaved by ACh to produce thiocholine. Thiocholine then reacts with 5,5’-dithiobis (2-nitrobenzoic acid) and gives rise to the yellow compound TNB. The absorbance at 412 nm has been quantified and directly relates to TNB formation and hence acetylcholinesterase activity (Gorun et al., 1997).

### Na^+^, K^+^-ATPase activity

Na^+^, K^+^-ATPase activity was assayed spectrophotometrically through the measurement of the ATP hydrolysis rate. To calculate the specific activity for Na⁺, K⁺-ATPase activity, Mg²⁺-dependent ATPase activity was deducted from total ATPase activity (Na⁺, K⁺, Mg²⁺-dependent). The absorbance of the resulting product at 640 nm was measured (Tsakiris et al., 2000).

### Monoamine oxidase (MAO) activity

Monoamine oxidase (MAO) activity was determined via the method described by Holt et al. (1997), where benzylamine is enzymatically converted to benzaldehyde. The formation of benzaldehyde was quantified by measuring its absorbance at 250 nm.

### Monoamine neurotransmitters

The concentrations of serotonin (5-HT), norepinephrine (NE), epinephrine (Epi), dopamine (DA), and 3,4-dihydroxyphenylacetic acid (DOPAC) were measured at 450 nm and expressed in ng/ml serum depending on reacting with specific reagent to form a color complex via radioenzymatic procedure (Da Prada and Zürcher, 1976).

### Statistical analysis

Data were presented as means ± standard error (SEM) using one-way analysis of variance (ANOVA) via the Statistical Package for Social Sciences (SPSS) program (version 27) to compare the means of different groups dependent variables. When a statistically significant P-value ( P ≤ 0.05) was achieved, post hoc tests via Duncan’s test were conducted to determine specific group differences.

### Histological Preparation

The brain tissues from all the experimental animal groups were dissected into small fragments and fixed in aqueous Bouin’s solution for 24 h. The samples were then processed via standard paraffin embedding techniques, following the histological procedures outlined by Bancroft and Gamble (2002). Sections of 4–6 μm thickness were cut, stained with hematoxylin and eosin (H&E), dehydrated in xylene, mounted with DPX, and coverslipped. Microscopic examination was conducted via an Olympus BX-40 compound light microscope equipped with a Panasonic CD-220 camera. Five sections from each group were evaluated to assess and grade the varied pathological alterations observed in the brain tissues using high power magnification (x400). The degree of neuronal degeneration and vacuolation were represented using a four-point semiquantitative scoring system that graded (0–4) as follow: (0) indicates no changes, (1) indicates a percentage area affected (< 10%), (2) indicates a percentage area affected (20–30%), (3) indicates a percentage region affected (40–60%), and (4) indicates more than 60% (Ibrahim Fouad and Ahmed, 2021).

### Immunohistochemical preparations

Immunohistochemical analysis was conducted on brain tissues from both control and treated rats. The specimens were fixed in cold neutral buffered formalin for 24 h. The expression of the Iba-1 protein, a marker of microglial activation, was detected via the avidin-biotin complex (ABC) technique (Kiernan, 2015).

#### Tissue processing

Paraffin sections were deparaffinized in xylene and subsequently rehydrated through a graded series of ethanol. Following PBS rinse and a rinse in distilled water, the endogenous peroxidase activity was blocked by 3% hydrogen peroxide.

#### Immunostaining

The sections were incubated with a primary Iba-1 antibody (Thermo Fisher Scientific, PA5-121836) at room temperature for 1–2 h and overnight at 4 °C. The sections were washed extensively with PBS and then incubated with a biotinylated secondary antibody and avidin-biotin complex (ABC) to visualize protein expression.

### Visualization and counterstaining

The tissue sections were subsequently stained with a chromogenic substrate for the revelation of immunoreactive sites. The tissue sections were dehydrated through a series of graded alcohols, and counterstained in Mayer’s hematoxylin and mounted in DPX for microscopic examination.

### Image analysis for immunohistochemistry

Immunohistochemical staining was conducted via mouse/IgG kappa. The stained sections were initially examined and imaged via a compound light microscope integrated with Leica Qwin 500 image analysis software (Leica Microsystems, Germany). A cell was marked as positive if it showed visible brown staining in the cytoplasm or around the membrane that stood out clearly from the background. To evaluate Iba-1 immunoreactivity, the proportion of Iba-1-positive cells within each field of view was quantified at 200× magnification. Measurements were performed via a standardized frame covering an area of 11,434.9 mm² across five fields. Then, the image analysis system was calibrated to convert pixel data into precise micrometer units. The quantification of Iba-1-positive cell areas in each field was carried out at the Department of Oral and Dental Pathology, Faculty of Dental Medicine for Girls, Al-Azhar University. The average area percentage of Iba-1-positive cells was then calculated for each experimental group to facilitate statistical analysis.

## Results

### Behavioral tests

**Fig. 2:**
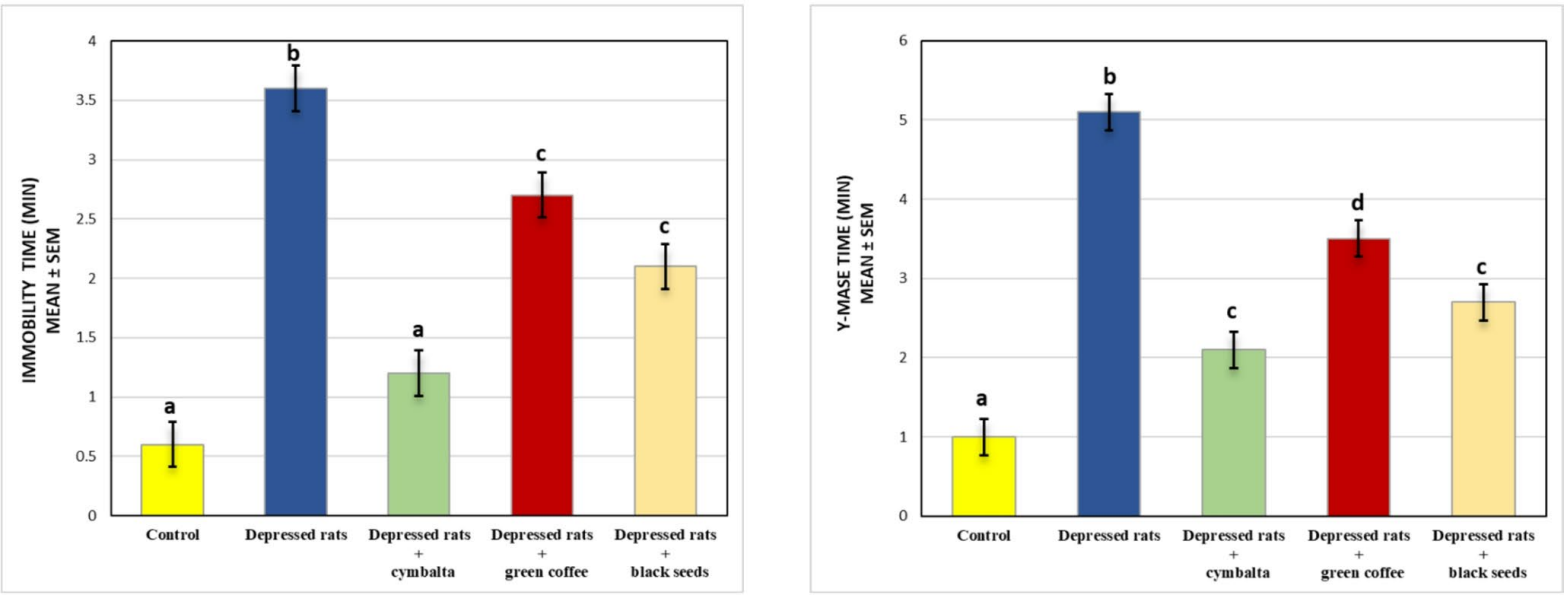
Behavioral Assessment of Cognitive and Immobility time Across Experimental Groups Using Forced Swim and Y-Maze TestsValues are presented as mean ± SEM. Columns sharing the same superscript letter are not significantly different from each other, while bars with different letters differ significantly (one-way ANOVA followed by Duncan post hoc test, *P ≤ 0.05*)

 In the forced swim and Y-maze tests, depressed rats exhibited significantly greater immobility and poorer memory performance than control animals did, confirming the stimulation of depressive-like behavior and cognitive deficits. The Cymbalta depressed group produced the most notable enhancement in both tests, with significantly reduced immobility and improved memory performance. The green coffee depressed group presented minor effects, whereas the black seed depressed group presented the lowest impact. These results emphasize Cymbalta’s greater antidepressant and cognitive-enhancing effects, whereas green coffee and black seeds offer limited but promising benefits, as demonstrated in Figure (2).

### Physiological results

Reserpine-induced depression significantly disrupted neurotransmitter levels, particularly by reducing DA; 5-HT; and Na^+^, K^+^ in addition to increasing DOPAC, MAO, ACh, Epi and NE. Cymbalta markedly restored these levels, whereas green coffee and black seeds resulted in moderate improvements, confirming their potential antidepressant effects through neurotransmitter modulation, as shown in (Table 1).

**Table 1:** Effects of Cymbalta, green coffee, and black seeds on the serum levels of DA, DOPAC, MAO, 5-HT, ACh, Epi, NE and Na^+^, K^+^ in the depressed rats induced by reserpine

Groups	Control	Depressed rats	% D	Depressed rats + Cymbalta	% D	Depressed rats + green coffee	% D	Depressed rats + black seeds	% D
DA (ng/mg/protein)	1.7^a^ ± 0.01	0.71^b^ ± 0.01	−58.2	1.5^c^ ± 0.01	−11.8	1.2^d^ ± 0.02	−29.4	1.4^e^ ± 0.01	−17.6
DOPAC (ng/mg/protein)	0.29^a^ ± 0.01	1.8^b^ ± 0.01	521	0.94^c^ ± 0.03	224	1.5^d^ ± 0.01	417	1.2^e^ ± 0.01	314
MAO (mU/mg/protein)	0.14^a^ ± 0.01	0.9^b^ ± 0.02	542.9	0.31^c^ ± 0.004	121.4	0.67^d^ ± 0.01	378.6	0.49^e^ ± 0.01	250
5-HT (ng/mg/protein)	2.5^a^ ± 0.01	0.82^b^ ± 0.01	−67.2	2.1^c^ ± 0.02	−16	1.4^d^ ± 0.01	−44	1.7^e^ ± 0.01	−32
ACh (ng/mg/protein)	0.47^a^ ± 0.01	2.4^b^ ± 0.01	411	1.6^c^ ± 0.01	240	2.1^d^ ± 0.01	347	1.9^e^ ± 0.02	304
Epi (pg/mg/protein)	0.44^a^ ± 0.01	1.9^b^ ± 0.17	332	1.1^c^ ± 0.01	150	1.6^d^ ± 0.01	264	1.4^d^ ± 0.01	218
NE (pg/mg/protein)	0.53^a^ ± 0.01	1.6^b^ ± 0.01	202	0.87^c^ ± 0.01	64.2	1.4^d^ ± 0.01	164	1.2^e^ ± 0.01	126
Na^+^/K^+^ (ng/mg/protein)	0.98^a^ ± 0.01	0.15^b^ ± 0.01	−84.7	0.75^c^ ± 0.01	−23.5	0.44^d^ ± 0.01	−55.1	0.66^e^ ± 0.01	−32.6

Values represent the Mean ± S.E.M.

Different letters in the same row represent statistically significant values (P-value ≤ 0.05) while the same letters represent statistically non-significant values.

% D: Percentage difference [(Treated value – Control Value)/Control Value] × 100

### Oxidative stress in the brain

Table 2 indicates that reserpine markedly increased oxidative stress, as shown by elevated MDA in addition to reduced GSH and NO levels in the brain tissues of the depressed rats. Cymbalta most effectively reversed these changes, while green coffee and black seeds also significantly improved the expression of oxidative stress markers, supporting their antioxidant potential in depressive conditions.

**Table 2:** Effects of Cymbalta, green coffee, and black seeds on the levels of of lipid peroxidation **(MDA)**, glutathione** (GSH) **and nitric oxide **(NO)** nitric oxide (**NO**), in the brain tissues of the depressed rats induced by reserpine.

Groups	Control	Depressed rat	% D	Depressed rats + Cymbalta	% D	Depressed rats + green coffee	% D	Depressed rats + black seeds	% D
MDA (n. mol/mg/protein)	0.82^a^ ± 0.01	5.1^b^ ± 0.1	522	1.9^c^ ± 0.02	132	3.7^d^ ± 0.08	351	2.5^e^ ± 0.09	205
GSH (m. mol/mg/protein)	6.2^a^ ± 0.09	1.4^b^ ± 0.04	−77.4	4.7^c^ ± 0.03	−24.2	2.4^d^ ± 0.12	−61.3	3.5^e^ ± 0.16	−43.5
NO (m. mol/mg/protein)	2.6^a^ ± 0.01	0.56^b^ ± 0.01	−78.5	1.8^c^ ± 0.01	−30.8	1.2^d^ ± 0.01	−53.8	1.4^e^ ± 0.01	−46.2

Values represent the Mean ± S.E.M.

Different letters in the same row represent statistically significant values (P-value ≤ 0.05) while the same letters represent statistically non-significant values

% D: Percentage difference [(Treated value – Control Value)/Control Value] × 100

### Histological results

**Fig. 3:**
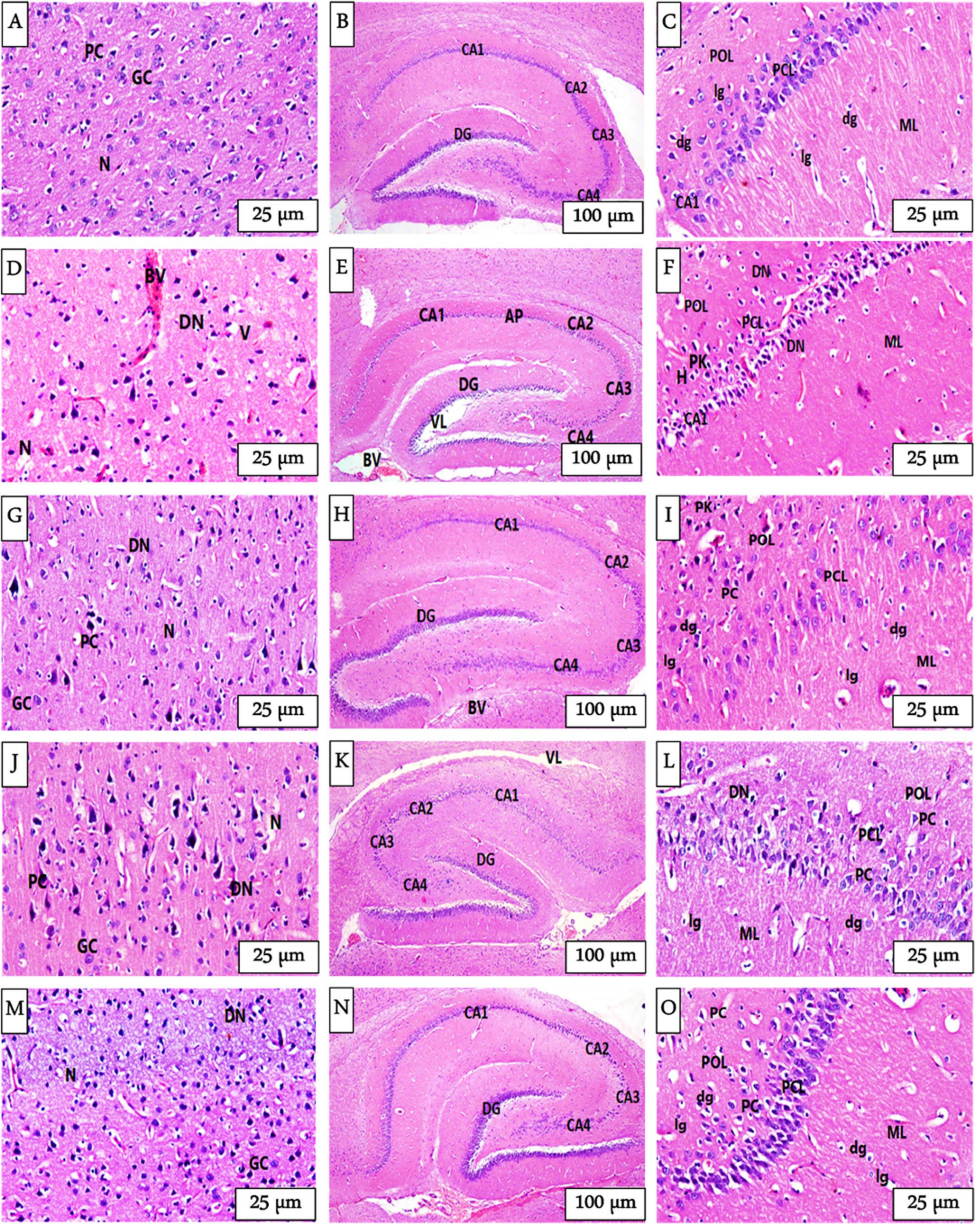
Photomicrographs of the brain regions; cerebral cortex and hippocampus of control and treated animal groups stained with H&E. **(A, B, C)**. The control group exhibits normal granular cells (**GC**), pyramidal cells (**PC**), and normal neuropil (**N**) in cortex. Hippocampus showing CA1, CA2, CA3, CA4 and dentate gyrus (**DG**) with well-defined layers; polymorphic layer (**POL**), pyramidal cell layer (**PCL**) and molecular layer (**ML**) besides, deeply (**dg**) and lightly (**lg**) stained nuclei of glial cells. Depressed rats **(D, E, F)** displayed irregular pyramidal cells with dark stain nuclei (**DN**), pericellular vacuoles (**V**), vacuolated neuropil (**N**), dilated and congested blood vessels (**BV**) in cortex. An apparent disruption in CA1, CA2, DG with dilated ventricular lumen (**VL**), apoptotic neurons (AP) and (BV). Cymbalta **(G, H, I)** showing near normal neurons in (GC), (PC), (CA1-CA4), (DG) with no dilatation of (VL). Low number of (DN), (PK) and vacuolated (N). Green coffee **(J, K, L)** demonstrated near normal (**GC**), (**PC**), (**DG**), CA1-CA4, (**dg**), (**lg**) with reduction in (**DN**), (**N**), (**VL**). Black seeds **(M, N, O)** similar to green coffee, near normal architecture in the two regions.

Histological examination of the cerebral cortex and hippocampus of control rats revealed normal neuroarchitecture. In the cerebral cortex, healthy granular cells and pyramidal neurons with triangular-shaped cell bodies were embedded within a well-preserved neuropil (Fig. 3 A). Similarly, in the hippocampus, the C-shaped structure was intact, with clearly defined CA1–CA4 regions and a prominent dentate gyrus (DG) (Fig. 3B). The CA1 region displayed a characteristic three-layered organization: a molecular layer (ML), a pyramidal cell layer (PCL), and a polymorphic layer (POL). Pyramidal neurons were closely packed in parallel rows with vesicular nuclei and prominent nucleoli, whereas glial cells were evenly distributed across the ML and POL (Fig. 3 C).

In contrast, rats treated with reserpine presented widespread histopathological deterioration in both the cortical and the hippocampal regions. The cerebral cortex exhibited numerous darkly stained degenerated neurons, irregular pyramidal cells with pyknotic and hyperchromatic nuclei, and extensive vacuolation of the neuropil. Congested and dilated blood vessels were also observed (Fig. 3D). In the hippocampus, there was significant thinning of the PCL, a dilated ventricular lumen, and degeneration of pyramidal neurons across the CA1, CA2, and DG regions (Fig. 3E). High magnification revealed pericellular halos, vacuolated neuropil, and numerous shrunken neurons with condensed chromatin. Glial cell nuclei appeared both intensely and lightly stained, indicating reactive gliosis (Fig. 3 F).

Treatment with Cymbalta led to partial restoration of neuronal integrity. In the cortex, granular and pyramidal cells presented improved morphology, with reduced numbers of degenerated neurons and partial resolution of neuropil vacuolation (Fig. 3G). In the hippocampus, the CA regions regained structural clarity with moderately restored PCL thickness (Fig. 3H). The pyramidal neurons appeared loosely arranged but retained vesicular nuclei. Occasional pyknotic neurons remained, although the overall cytoarchitecture was markedly improved (Fig. 3I).

Further histological improvement was observed in the rats treated with the green coffee extract. The cortex showed a more distinct and healthy neuronal structure, with granular and pyramidal cells maintaining vesicular nuclei and reducing neuronal degeneration. However, some pyramidal cells appeared flame-like with pointed ends (Fig. 3 J). The hippocampal architecture appeared nearly normal; the PCL was well defined, and the neuronal layers were orderly and structurally preserved. Only a few degenerated neurons remained, and mild vacuolation persisted in the neuropil. The glial distribution returned to near-control patterns, and the blood vessels appeared normal (Fig. 3 K & L).

Depressed rats treated with black seeds displayed a histology that closely resembled that of the control animals. In the cerebral cortex, most granular and pyramidal neurons appeared normal, with centrally located vesicular nuclei and minimal vacuolation of the neuropil (Fig. 3M). Similarly, in the hippocampus, the CA regions and DG presented intact laminar structures and neuronal layers (Fig. 3 N). The pyramidal cells exhibited well-defined nuclear features, and the neuropil showed minimal disruption, suggesting a strong neuroprotective or restorative effect of the extract against reserpine-induced degeneration (Fig. 3O). The histopathological lesion scores in the brain tissues of different groups are summarized in Table 3.

### Immunohistochemical results

**Fig. 4:**
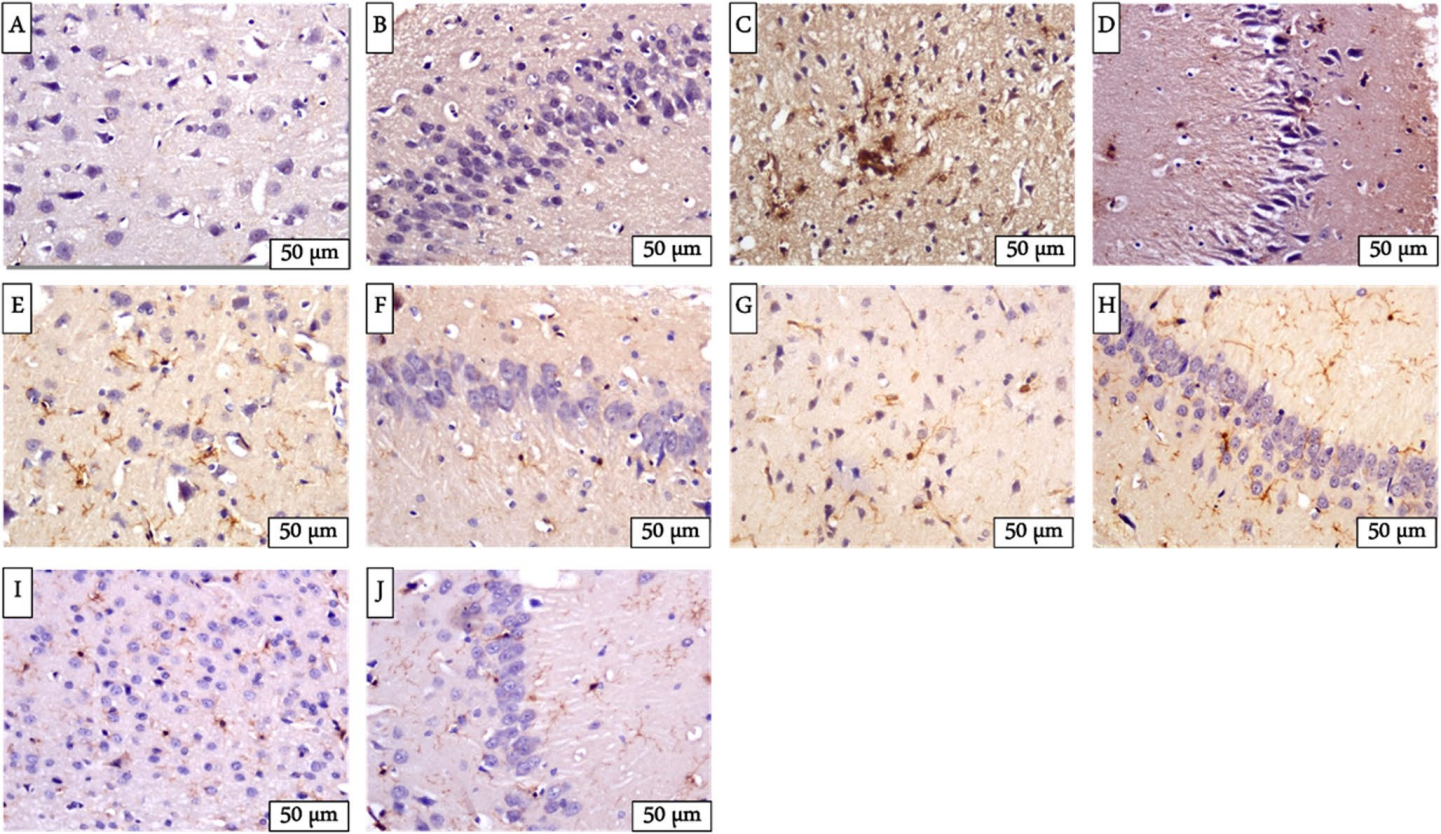
Immunohistochemical expression of Iba-1 in the cerebral cortex and hippocampus of control and treated rats **(A **&** B)** revealing mild Iba-1 immunoreactivity in control rats; **(C **&** D)** strong cytoplasmic and membranous immunostaining in reserpine-treated rats; **(E**&** F)** weak Iba-1 expression in the Cymbalta-treated group; **(G **&** H)** moderate immunoreactivity in green coffee-treated rats; **(I **&** J)** weak Iba-1 immunostaining in the brain tissue of rats treated with black seed extract

Immunohistochemical staining for ionized calcium-binding adaptor molecule 1 (Iba-1), a marker of activated microglia, revealed distinct patterns of expression across experimental groups. In the control group, brain sections demonstrated mild immunoreactivity to Iba-1 in both the cerebral cortex and hippocampus (Fig. 4 A & B).

In contrast, rats treated with reserpine presented noticeably strong Iba-1 immunoreactivity, particularly in the cytoplasm and membranes of cortical neurons. The hippocampal region in the same group also exhibited robust immunoreactivity (Fig. 4 C & D).

Compared with no treatment, treatment with Cymbalta (Fig. 4E & F) resulted in a marked reduction in Iba-1 immunoexpression. The cerebral cortex of Cymbalta-treated rats showed only mild cytoplasmic and membranous staining, whereas the hippocampus displayed similarly weak Iba-1 immunoreactivity.

In the group cotreated with green coffee and reserpine, Iba-1 immunoreactivity was moderate in the cerebral cortex, with clear staining localized to the membranes and cytoplasm of brain cells. The hippocampus of this group exhibited moderate to mild immunostaining (Fig. 4G & H).

Rats treated with black seed extract exhibited minimal Iba-1 immunoreactivity in both brain regions. The cerebral cortex showed weak and limited staining, whereas the hippocampus presented only weak immunoexpression of the Iba-1 protein was detected (Fig. 4I & J).

**Fig. 5:**
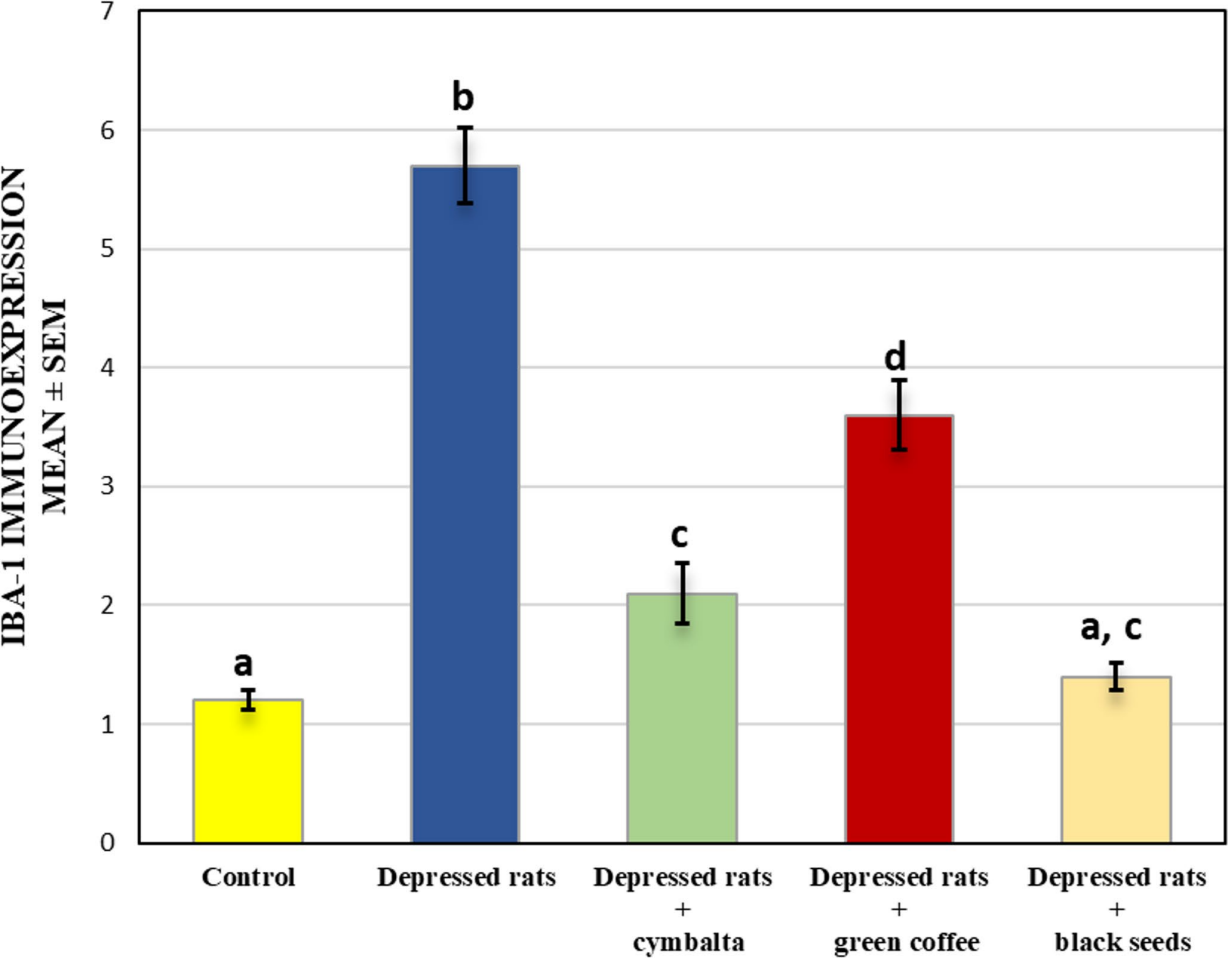
Proportion of microglial activation (Iba-1) in the brain tissues of the control and treated animal groups as determined by quantitative immunohistochemical image analysis. Values are presented as mean ± SEM. Columns sharing the same superscript letter are not significantly different from each other, while bars with different letters differ significantly (one-way ANOVA followed by Duncan post hoc test, P ≤ 0.05)

As shown in Fig. 5, brain tissue sections from reserpine-treated rats presented a significant increase (*P* ≤ 0.05) in Iba-1 immunoexpression compared with those from control rats. In contrast, tissues from Cymbalta-treated rats presented significantly lower Iba-1 immunoreactivity than did those from the depressed group, although the values remained greater than those of the control group. Compared with the reserpine group, the green coffee group also presented a moderate but statistically significant decrease in Iba-1 immunoexpression. On the other hand, compared with those from the depressed group, the brain tissues from the rats that were administered black seed extract presented a more pronounced reduction in Iba-1 levels, which approached the control values.

**Table 3:** Quantitative histopathological lesion scores in the brain tissues of all experimental groups

Histopathological lesion in the brain tissues	Control	Depressed rat	Depressed rats + Cymbalta	Depressed rats + green coffee	Depressed rats + black seeds
Neuronal Degeneration	0	3	2	1	1
Vacuolation	0	3	1	1	1
Microglial Activation	0	4	1	2	1

Values are presented as mean ± SEM. Columns sharing the same superscript letter are not significantly different from each other, while bars with different letters differ significantly (one-way ANOVA followed by Duncan post hoc test, *P* ≤ 0.05).

## Discussion

Depression, a complex mental illness characterized by persistent sadness and cognitive physical symptoms, significantly impacts quality of life and arises from genetic, biological, psychological, and environmental factors (Rana *et* al., 2020). Treatments such as Cymbalta increase 5-HT and NE levels but often cause side effects such as nausea and headaches (Alvarez-Mon et al., 2023). The safety and efficacy of natural alternatives such as green coffee and black seeds are gaining attention. Green coffee, which is rich in chlorogenic acids (CGAs), reduces oxidative stress and enhances neurogenesis (Socała et al., 2021), whereas the thymoquinone of black seeds modulates neurotransmitters and has neuroprotective effects (Samad et al., 2021), suggesting promising adjuncts or alternatives to conventional antidepressants.

The present study investigated the effects of Cymbalta, green coffee, and black seeds on neurotransmitter levels, oxidative stress markers, and neuroinflammation in a rat model of depression induced by reserpine. The results revealed significant alterations in the biochemical and immunohistochemical parameters across the treatment groups, providing insights into the mechanisms underlying their antidepressant and neuroprotective effects.

### Behavioral analysis

The results of the behavioral tests revealed significant differences in depression-like behavior and cognitive performance across all the experimental groups. In the FST, the depressed group presented the greatest immobility time, reflecting severe depressive-like behavior induced by reserpine, which is interpreted as a lack of motivation or escape-directed behavior, akin to helplessness (Samad et al., 2021). In contrast, Cymbalta significantly reduced immobility time by enhancing monoaminergic transmission, counteracting the monoamine-depleting effects of reserpine (Anand et al., 2025). Compared with those of depressed rats, the immobility times of green coffee and black seeds, although less pronounced than those of Cymbalta, are also reduced, suggesting that these substances have antidepressant effects (Alam et al., 2025).

In the Y-maze test, the depressed group presented the lowest spontaneous alternation percentage, indicating impaired spatial working memory. On the other hand, Cymbalta showed the most significant improvement, highlighting Cymbalta’s cognitive-enhancing effects. This result is in line with Meejuru et al. (2021), who noted an increase in memory activity. Green coffee and black seeds presented higher alternation percentages than depressed seeds did, suggesting cognitive benefits for these substances, but these percentages were still lower than those of Cymbalta. Imam-Fulani et al. (2022) reported an increase in the percentage of alternation of memory after green coffee administration. Additionally, Abo Mansour et al. (2023) reported that thymoquinone in black seeds enhances the cumulative percentage of spontaneous alteration.

Overall, the depression group displayed the most severe impairments, confirming the reserpine-induced depression model. The Cymbalta depressed group presented the most substantial improvements in both depressive-like behavior and cognitive function, followed by the green coffee and black seed groups. These findings suggest that while Cymbalta is highly effective in modulating behavior and cognitive function, green coffee and black seeds also offer beneficial benefits.

## Brain functions

The analysis of the results indicated a considerable difference between all experimental groups. The depressed group showed significant decreases in DA, 5-HT and Na⁺/K⁺ ATPase alongside increases in DOPAC, MAO and ACh since reserpine inhibits vesicular monoamine transporter 2 (VMAT2), the action of which prevents the neurotransmitters DA and 5-HT from being packaged inside synaptic vesicles. This causes an over-accumulation of cytoplasmic monoamines which can be metabolized by the action of the enzyme MAO. Therefore, the neurotransmitter levels in the synaptic cleft become decreased and contribute to depression (Strawbridge et al., 2023). The decreased concentration of DA as well as increased DOPAC, a principal metabolite of DA, reflects increased breakdown as well as turnover of dopamine. In response, the brain over-compensates by increasing the activity of MAO and thus affecting dopamine metabolism (Sanz-Novo et al., 2023). Na⁺/K⁺ ATPase levels decrease significantly, highlighting impaired ion regulation. This likely results from free radicals produced by the oxidative catabolism of monoamines (Khadrawy et al., 2024). Elevated ACh levels may be a compensatory response to a dopaminergic imbalance, particularly in areas such as the basal ganglia, involved in motor control and mood regulation, highlighting the complex effects of reserpine on neurotransmitter processes and its role in the biochemical mechanisms of depression (Calabresi et al., 1989). These results are similar to those obtained by Samad et al. (2021), who noted an increase in ACh levels after reserpine administration, and Abdel-Rasoul et al. (2023) reported that reserpine reduced the levels of DA and 5-HT.

According to previous studies (Antkiewicz-Michaluk et al., 2014; Strawbridge et al., 2023), reserpine reduces NE and Epi levels, but our findings revealed an increase in their levels, which, in turn to the depletion of brain NE and Epi, can trigger a compensatory reaction, resulting in increased release of neurotransmitters from the adrenal glands into the blood. Prolonged reserpine administration can further induce adaptation by augmenting the storage or release of NE and Epi in the peripheral system to compensate for decreased brain content (Fortin And Sundaresan, 1990; Veith et al., 1994).

In this study, Cymbalta demonstrated the most pronounced restoration of all brain function parameters, such as DA, 5-HT, and NE levels, as it inhibits the reuptake of 5-HT and NE. By blocking the reuptake of these neurotransmitters, Cymbalta increases their concentrations in the synaptic cleft, thereby enhancing serotonergic and noradrenergic neurotransmission (Stahl, 2022).

Green coffee and black seeds also enhanced neurotransmitter levels but less so than Cymbalta did. These results indicate that Cymbalta directly affects neurotransmitter reuptake, whereas green coffee and black seeds may act indirectly. Green coffee extract has been found to have antidepressant properties via modulating key monoaminergic systems besides antioxidant effects involved in depression. CGAs have an inhibitory effects on MAO that reducing the degradation of neurotransmitters which are reduced in depressive states. This preserves neurotransmitters, enhancing synaptic availability and mood regulation (Socała et al., 2021). Black seeds also have bioactive compounds; thymoquinone which have antioxidant, anti-inflammatory and neuroprotective properties (Imam et al., 2016). The changes found in neurotransmitter levels and enzymatic activity are via MAO inhibition, hypothalamic pituitary adrenal axis normalization, and neurotrophic support which modulate brain functions balance and improve the depressive symptoms (Pottoo et al., 2022).

## Oxidative Stress Markers

Role of oxidative stress in the pathophysiology of depression is crucial. The reserpine-induced depressed group showed high levels of MDA, a lipid peroxidation marker, and low levels of GSH, an endogenous antioxidant as reserpine an inhibitor of VMAT2, accumulates free cytosolic monoamines, leading to oxidative metabolism via MAO, generating reactive oxygen species (ROS) which elevated MDA and depleted GSH. NO levels decline due to its reaction with superoxide to form peroxynitrite, impairing neuronal signaling (Kuzay et al., 2022). Conversely, Cymbalta-treated depressed rat groups showed significantly lower levels of MDA and higher levels of GSH and NO, showing the possible protective effect of Cymbalta against oxidative stress (Ma et al., 2016; Zomkowski et al., 2012). Green coffee possesses potent antioxidant effects, which is consistent with previous studies showing its ability to scavenge free radicals that mitigate oxidative stress (Socała et al., 2021). Furthermore, black seeds which contain thymoquinone effectively reduce oxidative stress, supporting their role as natural antioxidants (Samad et al., 2021).

### Histological & immunohistochemical analysis

Histological examination of the cerebral cortex and hippocampus across different experimental groups revealed significant structural and cellular variations, highlighting the impact of depression and the potential restorative effects of pharmacological and natural interventions.

The control group showed normal histology of the cerebral cortex and hippocampus. The cerebral cortex showed well-defined and healthy neurons, and the hippocampus had a normal C-shaped structure and intact pyramidal layers. In contrast, the depressed group showed extensive degenerative changes. These changes involved the occurrence of dark degenerated neurons and vacuolated neuropil in addition to definitive features of apoptotic neurons and oxidative stress common under conditions of chronic stress and depressive states (Dobrek and Głowacka, 2023). Such damage reflects a compromise in neuronal plasticity and integrity and can compromise cognitive and emotional processing. Likewise, in the hippocampus, depressed rats showed hippocampal atrophy and dilated ventricles as well as apoptotic neurons. These changes reflect the cognitive impairments usually seen in depression as well as in stress-evoked neuronal damage (Duman And Aghajanian, 2012; Dobrek and Głowacka, 2023).

Treatment with Cymbalta restored both brain areas. In the cortex, the neural structures were maintained and less degenerated. Likewise, the atrophy lessened, and the pyramidal cell layer became better organized in the hippocampus. Cymbalta’s neuroprotective properties stem from the fact that it increases the levels of monoamines, restores deficits in neuroplasticity, and triggers anti-inflammatory pathways (Piao et al., 2023).

The green coffee administration proved significant neuroprotective effects, primarily on restoring the neuron’s structure. In the cerebral cortex, green coffee treatment restored almost normal neuron architecture and reduced degenerative markers like pyknotic nuclei and neuropil vacuolation significantly. Also, in the hippocampus, green coffee maintained the pyramidal layers’ integrity and decreased the extent of neuronal apoptosis and structural disordering. All these findings could be linked to the high chlorogenic acid content in green coffee that possesses anti-inflammatory and antioxidant properties. This compound supports neuronal survival and synaptic integrity, protecting the brain from stress-induced damage (dos Santos et al., 2023).

Additionally, black seeds demonstrated significant neuroprotective effects. In the cerebral cortex, treatment resulted in clear preservation of neuronal organization. The presence of degenerative features was markedly reduced. In the hippocampus, black seeds prevent hippocampal atrophy and support the maintenance of cellular architecture. These beneficial effects are attributed to bioactive compounds in black seeds, such as thymoquinone, which promote neuronal repair, reduce inflammation, and enhance histological recovery (Küçük and Baskın, 2024). These findings align with earlier findings that both pharmacological antidepressants and natural compounds can synergistically reduce inflammation and stimulate neuronal regeneration (Ahmad et al., 2013).

Neuroinflammation, which is characterized by microglial activation, is another hallmark of depression. Immunohistochemical analysis of Iba-1 expression revealed significant microglial activation in the depressed group, which was attenuated by all the treatments. This aligns with the findings of Yirmiya et al. (2015), who linked depression to increased microglial activity. Cymbalta showed the most substantial reduction in Iba-1 expression, suggesting that it has anti-inflammatory properties. This finding is consistent with that of Kim et al. (2021). Green coffee and black seeds also reduced neuroinflammation, although less effectively than did Cymbalta. This reduction returns to the inhibition of inflammatory cytokines, thereby preserving neuroplasticity and limiting hippocampal and cortical damage. These results are in agreement with previous studies (Prieur And Jadavji, 2019; Abo Mansour et al., 2023) that demonstrated the anti-inflammatory effects of CGAs and thymoquinone. The present results prove that antidepressants and natural compounds can attenuate neuroinflammation.

Overall, the results highlight the neuroprotective and anti-inflammatory effects of Cymbalta, green coffee and black seeds in a depressed animal model, supporting their potential therapeutic roles in mitigating depression- related to neuronal damage and inflammation.

## Conclusion

This study provides evidence that Duloxetine (Cymbalta), green coffee, and black seeds mitigate the neurochemical and oxidative stress-related abnormalities associated with depression. Cymbalta had the most significant antidepressant effects, followed by black seeds and green coffee , which demonstrated moderate efficacy. The antidepressant mechanisms of these agents involve the regulation of neurotransmitters, a reduction in oxidative stress, and the attenuation of neuroinflammation. These findings suggest that natural compounds such as green coffee and black seeds may serve as adjunctive or alternative treatments for depression. Further research is needed to explore their therapeutic potential in clinical settings.

## Data Availability

Data is provided within the manuscript.

## References

[CR1] Abdel-Rahman M, Arafa NMS, El-Khadragy MF, Kassab RB (2013) The neuroprotective role of Nigella sativa extract on Ciprofloxacin and Pentylenetetrazole treated rats. AJPP 7(24):6601670. 10.5897/AJPP12.897

[CR2] Abdel-Rasoul A, Saleh N, Hosny E et al (2023) Cardamom oil ameliorates behavioral and neuropathological disorders in a rat model of depression induced by reserpine. J Ethnopharmacol 308:116254. 10.1016/j.jep.2023.116254 36781058 10.1016/j.jep.2023.116254

[CR3] Abo Mansour HE, Elberri AI, Ghoneim ME et al (2023) The potential neuroprotective effect of thymoquinone on Scopolamine-induced in vivo Alzheimer’s disease-like condition: mechanistic insights. Molecules 28(18):6566. 10.3390/molecules28186566 37764343 10.3390/molecules28186566 PMC10534545

[CR4] Ahmad A, Husain A, Mujeeb M et al (2013) A review on therapeutic potential of *Nigella sativa*: a miracle herb. Asian Pac J Trop Biomed 3(5):337–352. 10.1016/S2221-1691(13)60075-1 23646296 10.1016/S2221-1691(13)60075-1 PMC3642442

[CR5] Alam MZ, Bagabir HA, Zaher MAF et al (2025) Black seed Oil-Based Curcumin nanoformulations ameliorated Cuprizone-Induced demyelination in the mouse hippocampus. Mol Neurobiol 62:604–625. 10.1007/s12035-024-04310-5 38890237 10.1007/s12035-024-04310-5

[CR6] Alayunt NÖ, Kayaoğlu Y, Yılmaz M, Üstündağ B (2023) Nigella sativa oil improves oxidative Stress, inflammation and changes in neurotransmitter structures in Fructose-Induced metabolic syndrome. Euroasia J Math Eng Nat Med Sci 10(29):128–138. 10.5281/zenodo.8416168

[CR7] Ali DH, Hegazy HG, Ali EHA et al (2025) *Ginkgo biloba* L. leaf extract (Egb 761) alleviates reserpine-induced depression-like symptoms in aged rats by enhancing serotonin/norepinephrine levels and reducing oxidative/nitrosative stress. Naunyn-Schmiedebergs Arch Pharmacol. 10.1007/s00210-025-03972-9 40100376 10.1007/s00210-025-03972-9 PMC12449422

[CR8] Alvarez-Mon MA, García-Montero C, Fraile-Martinez O et al (2023) Current opinions about the use of duloxetine: results from a survey aimed at psychiatrists. Brain Sci 13(2):333. 10.3390/brainsci13020333 36831876 10.3390/brainsci13020333 PMC9953910

[CR9] Anand J, Dhami K, Semwal P, Rai N (2025) Antidepression agents in Himalayan grains, beans, and nuts. Functional foods and nutraceuticals in metabolic and Non-Communicable disorders (Chap. 7). CRC. 10.1201/9781032717975-7

[CR10] Antkiewicz-Michaluk L, Wąsik A, Możdżeń E et al (2014) Antidepressant-like effect of tetrahydroisoquinoline amines in the animal model of depressive disorder induced by repeated administration of a low dose of reserpine: behavioral and neurochemical studies in the rat. Neurotox Res 26(1):85–98. 10.1007/s12640-013-9454-8 24407488 10.1007/s12640-013-9454-8 PMC4035545

[CR11] Bancroft J, Gamble M (2002) Theory and practice of histological techniques, 5th edn. Churchill Livingstone, London

[CR12] Beutler E, Duron O, Kelly OM (1963) Improved method for the determination of blood glutathione. J Lab Clin Med 61:882–888 13967893

[CR13] Bhatt S, Devadoss T, Jha N (2023) Targeting inflammation: a potential approach for the treatment of depression. Metab Brain Dis 38(1):45–59. 10.1007/s11011-022-01095-1 36239867 10.1007/s11011-022-01095-1

[CR14] Calabresi P, Stefan A, Mercuri NB, Bernardi G (1989) Acetylcholine-dopamine balance in striatum: Is it still a target for antiparkinsonian therapy? In: Frotscher, M., Misgeld, U. (eds) Central Cholinergic Synaptic Transmission. Experientia Supplementum, vol 57. Birkhäuser Basel. 10.1007/978-3-0348-9138-7_31 10.1007/978-3-0348-9138-7_31 2533102

[CR15] Da Prada M, Zürcher G (1976) Simultaneous radioenzymatic determination of plasma and tissue adrenaline, noradrenaline and dopamine within the femtomole range. Life Sci 19(8):1161–1174. 10.1016/0024-3205(76)90251-4 994720 10.1016/0024-3205(76)90251-4

[CR16] Davies DL, Shepherd M (1955) Reserpine in the treatment of anxious and depressed patients. Lancet (London England) 269(6881):117–120. 10.1016/s0140-6736(55)92118-8 14392947 10.1016/s0140-6736(55)92118-8

[CR17] Dobrek L, Głowacka K (2023) Depression and its phytopharmacotherapy—a narrative review. Int J Mol Sci 24(5):4772. 10.3390/ijms24054772 36902200 10.3390/ijms24054772 PMC10003400

[CR18] dos Santos FKF, Silva IGCB, Nunes Filho AL (2023) Coffee and Folk Medicine: Mechanisms and Activities. In V. F. da Veiga Júnior (Ed.), Coffee: From Cultivation to Health Benefits (Chap. 3). 10.1201/9781003323969-3

[CR19] Duman RS, Aghajanian GK (2012) Synaptic dysfunction in depression: potential therapeutic targets. Science 338(6103):68–72. 10.1126/science.1222939 23042884 10.1126/science.1222939 PMC4424898

[CR20] Fernandes MYD, Dobrachinski F, Silva HB et al (2021) Neuromodulation and neuroprotective effects of chlorogenic acids in excitatory synapses of mouse hippocampal slices. Sci Rep 11(1):10488. 10.1038/s41598-021-89964-0 34006978 10.1038/s41598-021-89964-0 PMC8131611

[CR21] Fortin TL, Sundaresan PR (1990) Reserpine up-regulation of rat renal cortical beta adrenergic receptors is independent of its effect on the sympathetic nervous system. J Pharmacol Exp Ther 253(3):913–920 1972754

[CR22] Gorun V, Proinov I, Baltescu V et al (1997) Modified Ellman procedure for assay of cholinesterase in crude-enzymatic preparations. Anal Biochem 86:324–326 10.1016/0003-2697(78)90350-0 655393

[CR23] Holt A, Sharman DS, Baker GB, Palcic MM (1997) A continuous spectrophotometric assay for monoamine oxidase and related enzymes in tissue homogenates. Anal Biochem 244:384–392 9025956 10.1006/abio.1996.9911

[CR24] Hutachok N, Koonyosying P, Pankasemsuk T et al (2021) Chemical analysis, toxicity study, and free-radical scavenging and iron-binding assays involving coffee (*Coffea arabica*) extracts. Molecules 26(14):4169. 10.3390/molecules26144169 34299444 10.3390/molecules26144169 PMC8304909

[CR25] Ibrahim Fouad G, Ahmed KA (2021) Neuroprotective potential of berberine against doxorubicin-induced toxicity in rat’s brain. Neurochem Res 46(12):3247–3263. 10.1007/s11064-021-03428-5 34403065 10.1007/s11064-021-03428-5

[CR26] Imam A, Ajao M, Ajibola M et al (2016) Black seed oil reversed scopolamine-induced alzheimer and cortico hippocampal neural alterations in male Wistar rats. Bull Fac Pharm Cairo Univ 54(1):1–106. 10.1016/j.bfopcu.2015.12.005

[CR27] Imam-Fulani AO, Ogungbemi OJ, Olajide LO et al (2022) Caffeine and *camellia sinensis* enhance cognition and decrease acetylcholinesterase activity in scopolamine-induced memory loss in female Swiss mice. Ann Med Physiol 6(2):8–15. 10.23921/amp.2022v6i2.00062

[CR28] Kato D, Suto T, Obata H, Saito S (2022) The efficacy of duloxetine depends on spinal cholinergic plasticity in neuropathic pain model rats. IBRO Neurosci Rep 12:188–196. 10.1016/j.ibneur.2022.02.004 35243478 10.1016/j.ibneur.2022.02.004 PMC8881419

[CR29] Khadrawy YA, Sawie HG, Hosny EN, Mourad HH (2018) Assessment of the antidepressant effect of caffeine using rat model of depression induced by reserpine. Bull Natl Res Cent 42 – 36. 10.1186/s42269-018-0034-1

[CR30] Khadrawy YA, Hosny EN, Eldein Mohamed HS (2024) Assessment of the neuroprotective effect of green synthesized iron oxide nanoparticles capped with Curcumin against a rat model of parkinson’s disease. Iran J Basic Med Sci 27(1):81–89. 10.22038/IJBMS.2023.73124.15892 38164480 10.22038/IJBMS.2023.73124.15892 PMC10722487

[CR31] Kiernan J (2015) Histological and histochemical methods: theory and practice, 4th edn. Scion Publishing, Banbury, pp 454–548

[CR32] Kim J, Lee JH, Song Y et al (2021) Application of PLGA nanoparticles to enhance the action of Duloxetine on microglia in neuropathic pain. Biomater Sci 9(18):6238–6251. 10.1039/D1BM00486G 10.1039/d1bm00486g 34378557

[CR33] Küçük N, Baskın V (2024) Nigella sativa and Brain. In E. İzol (Ed.), Neurological Diseases and Treatments in Terms of Biochemistry, pp: 174. 10.69860/nobel.9786053359357

[CR34] Kuzay D, Dileköz E, Özer Ç (2022) Effects of thymoquinone in a rat model of reserpine-induced depression. Braz J Pharm Sci 58:e19847. 10.1590/s2175-97902022e19847

[CR35] Ma J, Zhang Y, Liu H (2016) Oxidative stress and mitochondrial dysfunction in depression. Prog Neuropsychopharmacol Biol Psychiatry 69:23–35. 10.1016/j.pnpbp.2016.02.005

[CR36] Meejuru GF, Somavarapu A, Danduga RCSR et al (2021) Protective effects of duloxetine against chronic immobilization stress-induced anxiety, depression, cognitive impairment and neurodegeneration in mice. J Pharm Pharmacol 73(4):522–534. 10.1093/jpp/rgaa003 33793839 10.1093/jpp/rgaa003

[CR37] Molska GR, Paula-freire LIG, Sakalem ME et al (2021) Green coffee extract attenuates Parkinson’s-related behaviors in animal models. An Acad Bras Cienc 93:e20210481. 10.1590/0001-3765202120210481 34730624 10.1590/0001-3765202120210481

[CR38] Montgomery HAC, Dymock JF (1961) The determination of nitrite in water. Analyst 6:414–416

[CR39] Mosalam EM, Atya SM, Mesbah NM, Allam S, Mehanna ET (2025) Neuroprotective effects of cilomilast and chlorogenic acid against Scopolamine-induced memory deficits via modulation of the cAMP/PKA–CREB–BDNF pathway. Int J Mol Sci 26:3108. 10.3390/ijms26073108 40243772 10.3390/ijms26073108 PMC11988773

[CR40] Nakamura J, Kim E, Rentscher K (2022) Early-life stress, depressive symptoms, and inflammation: the role of social factors. Aging Ment Health 26(4):843–851. 10.1080/13607863.2021.1876636 33502257 10.1080/13607863.2021.1876636 PMC8313624

[CR41] Nasim N, Sandeep I, Mohanty S (2022) Plant-derived natural products for drug discovery: current approaches and prospects. Nucleus (Calcutta) 65(3):399–411. 10.1007/s13237-022-00405-3 36276225 10.1007/s13237-022-00405-3 PMC9579558

[CR42] Piao J, Wang Y, Zhang T et al (2023) Antidepressant-like effects of representative types of food and their possible mechanisms. Molecules 28(19):6992. 10.3390/molecules28196992 37836833 10.3390/molecules28196992 PMC10574116

[CR43] Porsolt RD, Bertin A, Jalfre M (1977) Behavioral despair in mice: a primary screening test for antidepressants. Arch Int Pharmacodyn Ther 229(2):327–336 596982

[CR44] Pottoo FH, Ibrahim AM, Alammar A (2022) Thymoquinone: review of its potential in the treatment of neurological diseases. Pharmaceuticals 15(4):408. 10.3390/ph15040408 35455405 10.3390/ph15040408 PMC9026861

[CR45] Prieur E, Jadavji N (2019) Assessing spatial working memory using the spontaneous alternation Y-maze test in aged male mice. Bio-protocol 9(3):e3162. 10.21769/BioProtoc.3162 33654968 10.21769/BioProtoc.3162 PMC7854095

[CR46] Rana I, Khan N, Ansari M et al (2020) Solid lipid nanoparticles-mediated enhanced antidepressant activity of Duloxetine in lipopolysaccharide-induced depressive model. Colloids Surf B Biointerfaces 194:111209. 10.1016/j.colsurfb.2020.111209 32599505 10.1016/j.colsurfb.2020.111209

[CR47] Remali J, Aizat W (2024) Medicinal plants and plant-based traditional medicine: alternative treatments for depression and their potential mechanisms of action. Heliyon 10:e38986. 10.1016/j.heliyon.2024.e38986 39640650 10.1016/j.heliyon.2024.e38986 PMC11620067

[CR48] Ruiz-Larrea M, Leal A, Liza M et al (1994) Antioxidant effects of estradiol and 2-hydroxyestradiol on iron-induced lipid peroxidation of rat liver microsomes. Steroids 59:383–388. 10.1016/0039-128X(94)90006-X 7940617 10.1016/0039-128x(94)90006-x

[CR49] Ruiz N, Del Ángel D, Brizuela N et al (2022) Inflammatory process and immune system in major depressive disorder. Int J Neuropsychopharmacol 25(1):46–53. 10.1093/ijnp/pyab072 34724041 10.1093/ijnp/pyab072 PMC8756095

[CR50] Samad N, Manzoor N, Muneer Z (2021) Reserpine-induced altered neuro-behavioral, biochemical and histopathological assessments prevent by enhanced antioxidant defence system of thymoquinone in mice. Metab Brain Dis 36:2535–2552. 10.1007/s11011-021-00789-2 34309746 10.1007/s11011-021-00789-2

[CR51] Sanz-Novo M, Kolesniková L, Insausti A et al (2023) A journey across dopamine metabolism: A rotational study of DOPAC. Spectrochim Acta - A: Mol Biomol Spectrosc 290:1386–1425. 10.1016/j.saa.2022.122303 10.1016/j.saa.2022.122303 36608514

[CR52] Socała K, Szopa A, Serefko A et al (2021) Neuroprotective effects of coffee bioactive compounds: A review. Int J Mol Sci 22(1):107. 10.3390/ijms22010107 10.3390/ijms22010107 PMC7795778 33374338

[CR53] Stahl S (2022) Stahl’s essential psychopharmacology: prescriber’s guide. Cambridge University Press, Cambridge

[CR54] Strawbridge R, Javed RR, Cave J et al (2023) The effects of reserpine on depression: a systematic review. J Psychopharmacol 37(3):248–260. 10.1177/02698811221115762 36000248 10.1177/02698811221115762 PMC10076328

[CR55] Tsakiris S, Angelogianni P, Schulpis KH, Stavridis JC (2000) Protective effect of L-phenylalanine on rat brain acetylcholinesterase inhibition induced by free radicals. Clin Biochem 33:103–106 10751587 10.1016/s0009-9120(99)00090-9

[CR56] Veith RC, Lewis N, Linares OA et al (1994) Sympathetic nervous system activity in major depression. Arch Gen Psychiatry 51(5):411–422. 10.1001/archpsyc.1994.03950050071008 8179465 10.1001/archpsyc.1994.03950050071008

[CR57] Yirmiya R, Rimmerman N, Reshef R (2015) Depression as a microglial disease. Trends Neurosci 38(10):637–658. 10.1016/j.tins.2015.08.001 26442697 10.1016/j.tins.2015.08.001

[CR58] Yusni Y, Yusuf H (2022) The effect of green coffee on blood pressure, liver and kidney functions in obese model rats. Open Access Maced J Med Sci 10(A):346–351. 10.3889/oamjms.2022.8134

[CR59] Zomkowski AD, Engel D, Cunha MP et al (2012) The role of the NMDA receptors and l-arginine-nitric oxide-cyclic guanosine monophosphate pathway in the antidepressant-like effect of Duloxetine in the forced swimming test. Pharmacol Biochem Behav 103(2):408–417. 10.1016/j.pbb.2012.09.011 23010381 10.1016/j.pbb.2012.09.011

[CR60] Zygler A, Słomi´nska M, Namie´snik J (2012) Soxhlet extraction and new developments such as Soxtec. In: Ed PJ (ed) Comprehensive sampling and sample preparation: analytical techniques for scientists, 1st edn. Academic: London, UK, pp 65–82. 10.1016/B978-0-12-381373-2.00037-5

